# Supplementary material for: MetaRibo-Seq measures translation in microbiomes
Source: Nat Commun. 2020 Jun 29;11:3268. doi: 10.1038/s41467-020-17081-z (PMC7324362; doi:10.1038/s41467-020-17081-z)
Supplement: Supplementary file 10 — Supplementary Data 7 [file 41467_2020_17081_MOESM10_ESM.zip › File2/Confidence_High_Taxonomy/245680_out.krona.html]

Javascript must be enabled to view this page.

members
magnitude
magnitudeUnassigned
count
unassigned
taxon
rank

245680\_out

16


SRS020622\_contig\_number\_2665
superkingdom
9
2
1

phylum
4
1239

186801
4
class

186802
4
order

species

SRS100021\_contig\_number\_11477
172733
1

2
31979
family

genus
1485
1

species

SRS050026\_contig\_number\_12323
59620
1

genus
1
580596

species

SRS078665\_contig\_number\_contig-100\_128.76961
1193532
1

1
2109688
species

SRS142712\_contig\_number\_5017

4
1224
phylum

28211
3
class

order
1
204441

family
1
41295

1543705
1
genus

species

SRS1041138\_contig\_number\_contig-100\_8438.42440
1
28077

order
2
204455

2
1674811
species

SRS051610\_contig\_number\_8196SRS146764\_contig\_number\_27298

subphylum
1
68525

28221
1
class

order
1
213115

194924
1
family


SRS1041091\_contig\_number\_contig-100\_16365.16366
genus
1
35832

6

SRS017433\_contig\_number\_contig-100\_22754.61148SRS049402\_contig\_number\_contig-100\_4015.104630SRS054590\_contig\_number\_contig-100\_13343.13343SRS058770\_contig\_number\_contig-100\_22370.22370SRS064645\_contig\_number\_contig-100\_16953.16953SRS1041091\_contig\_number\_contig-100\_24943.60375

10239
1
superkingdom

order
28883
1

10699
1
family

1
2500567
species

SRS142980\_contig\_number\_contig-100\_3267.3268
